# Repression of Inappropriate Gene Expression in the Vertebrate Embryonic Ectoderm

**DOI:** 10.3390/genes10110895

**Published:** 2019-11-06

**Authors:** Shoshana Reich, Daniel C. Weinstein

**Affiliations:** 1 PhD Program in Biology, The Graduate Center, The City University of New York, New York, NY 10016, USA; 2 Department of Biology, Queens College, The City University of New York, Queens, NY 11367, USA

**Keywords:** *Xenopus*, development, gastrulation, ectoderm, gene regulation, pluripotency

## Abstract

During vertebrate embryogenesis, precise regulation of gene expression is crucial for proper cell fate determination. Much of what we know about vertebrate development has been gleaned from experiments performed on embryos of the amphibian *Xenopus laevis*; this review will focus primarily on studies of this model organism. An early critical step during vertebrate development is the formation of the three primary germ layers—ectoderm, mesoderm, and endoderm—which emerge during the process of gastrulation. While much attention has been focused on the induction of mesoderm and endoderm, it has become clear that differentiation of the ectoderm involves more than the simple absence of inductive cues; rather, it additionally requires the inhibition of mesendoderm-promoting genes. This review aims to summarize our current understanding of the various inhibitors of inappropriate gene expression in the presumptive ectoderm.

## 1. Germ Layer Formation

Triploblastic organisms form three germ layers—endoderm, mesoderm, and ectoderm—which give rise to all tissue types in the adult organism with the exception of the germ cells. Elucidation of the precise regulation of the mechanisms that specify cell fate in development is of the utmost importance [1]. Understanding the regulation of cell fate specification has far-reaching implications for various fields of study, including pluripotency, cancer, and gene regulation. While extensive work has identified many genes necessary for inducing cell fates during embryogenesis, it has become apparent that factors that prevent inappropriate germ layer expression are also crucial for normal development. Utilizing data primarily from experiments performed in *Xenopus laevis*, this review will discuss the restriction of mesodermal and endodermal gene expression in the ectoderm of developing vertebrate embryos, and the multiple factors that regulate this process.

## 2. Mesendoderm Induction 

**Figure 1:**
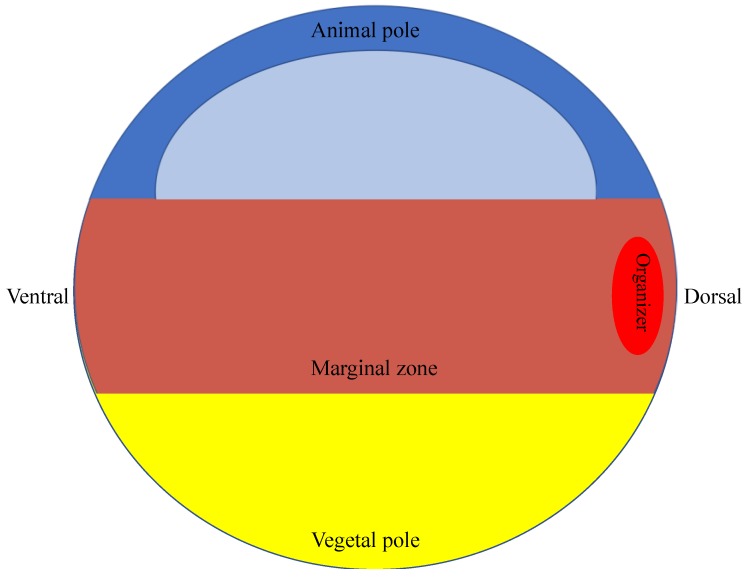
Diagram of *Xenopus laevis* embryo during early gastrulation. During gastrulation, the three primary germ layers, endoderm, mesoderm, and ectoderm, begin to differentiate. The vegetal pole refers to the lower hemisphere of the embryo and will give rise to the endoderm. The marginal zone refers to the equatorial region of the embryo between the animal and vegetal poles and will give rise to the mesoderm. The mesoderm contains a dorsal organizer region which secretes Bone Morphogenetic Protein (BMP) antagonists. The animal pole refers to the upper hemisphere of the embryo which will give rise to the ectoderm. The drawing of the cavity in the animal hemisphere depicts the fluid-filled blastocoel. As described in the text of the review, *foxI1e*, along with many other germ layer-enriched factors, is expressed in the cells of the animal pole. *chordin* and *goosecoid* are expressed in the dorsal marginal zone. *wnt8* is expressed ventrolaterally and *brachyury* is expressed throughout the marginal zone. VegT, an activator of *nodal* and *nodal-like* genes, is expressed in the cells of the vegetal pole.

During late blastula stages, the animal pole of a developing *Xenopus laevis* embryo consists of a pluripotent population of cells that can be induced to differentiate into various cell fates (Figure 1) [2]. To describe the mechanisms through which mesendoderm is suppressed in the amphibian animal pole, it is important that we first outline the pathways that specify mesendoderm induction. Mesendoderm refers to a transient, precursor cell population from which both mesoderm and endoderm emerge during gastrulation, and the molecular pathways that govern the formation of mesoderm and endoderm overlap to some extent [3]. As we describe below, many mesendodermal suppressors function by inhibiting the intracellular signal transduction pathways that specify mesendodermal induction. In the amphibian embryo, the differentiation of the germ layers initiates when VegT, a maternally supplied factor, stimulates expression of transcription factors, such as Bix4, to specify cells located in the vegetal pole to differentiate into endoderm (Figure 1) [4,5,6]. VegT also activates *nodal* and *nodal-related* gene expression; these transcripts encode proteins that induce cells in the region above the vegetal pole, called the marginal zone, to differentiate as mesoderm (Figure 1) [6,7]. The induction of mesoderm via the Activin/Nodal signaling pathway is known to be conserved across vertebrate species including zebrafish, *Xenopus laevis*, chicken, and mouse [8].

Activin/Nodal signaling initiates when Nodal, Nodal-like, and other related Transforming Growth Factor beta (TGFβ) ligands bind to the type II TGFβ receptor, which subsequently phosphorylates the type I receptor [9]. The type I and type II receptors then form a heterotetrameric complex, containing two of each receptor type. The activated type I receptor phosphorylates the receptor-activated Smads (R-Smads), Smad2, and Smad3. Once phosphorylated, Smad2 and Smad3 form a heteromeric complex with Smad4. This complex then translocates from the cytoplasm to the nucleus and, along with many associated proteins such as FoxHI, CREB binding protein, and Mixer, mediates the transcription of target genes [10,11,12]. Immediate-early targets of the Smad2/Smad4 complex include, among others, *goosecoid* and *mix.2* [10,11,13,14]. Through this signaling pathway, Activin/Nodal ligands induce mesoderm during gastrulation. 

In addition to the Activin/Nodal TGFβ pathway, several additional pathways are integral to mesoderm induction and maintenance in the developing embryo. For example, Fibroblast Growth Factor (FGF) signaling is required for the maintenance of mesoderm during gastrulation [15,16,17]. FGF and Brachyury function through an autocatalytic loop; FGF induces expression of *brachyury*, which in turn induces expression of e*FGF* [18]. Brachyury, a T-box transcription factor, is an immediate-early response to mesoderm induction and functions as an activator to turn on additional mesodermal genes (Figure 1) [19,20,21]. Additionally, β-catenin stabilization is necessary for proper FGF signaling in the prospective mesoderm during gastrulation [22,23].

FGF induces mesoderm through various downstream signaling mechanisms. FGF signaling leads to phosphorylation of the ERK mitogen-activated protein kinase (MAPK) pathway, which subsequently leads to phosphorylation of P53 [24,25]. Once phosphorylated, P53 physically associates with phosphorylated Smad2 to induce the expression of mesodermal genes [26,27]. Studies in mouse and mammalian cell culture show that independent from P53 phosphorylation, ERK also activates expression of many factors critical for mesodermal maintenance [28,29]. For example, ERK induces expression of Egr1, a transcription factor that regulates expression of FGF target genes [30]. 

## 3. Differentiation and Patterning of Ectoderm

The cells of the ectoderm give rise to several distinct tissue types. Ventral ectoderm differentiates into epidermal tissue, while neural tissue forms from the dorsal ectoderm; cells at the border between these two populations develop into the sensory placodes and neural crest [31,32]. During development, Bone Morphogenetic Protein (BMP) signaling gradients regulate dorsal/ventral patterning of the mesoderm [25]. BMP signaling has been shown to also be critical for ectodermal patterning [33]. Studies have shown that an abundance of BMP-4, initially widely expressed throughout the blastula, ventralizes the ectoderm which then differentiates into epidermis [33]. The Spemann organizer secretes multiple BMP antagonists that inhibit BMP signaling dorsally and allow dorsal ectodermal cells to adopt their “default” fate, neural tissue; when BMP signaling is inhibited throughout the prospective ectoderm, all ectodermal cells differentiate into neural tissue [34,35,36,37,38]. Classical studies suggest that the ectoderm forms because the cell population in the animal pole of the embryo does not receive inducing signals from the endoderm/mesoderm [25]. Recent work, however, has demonstrated that there are proteins expressed in the ectoderm necessary for active repression/restriction of mesodermal and endodermal fates. Below, we will describe the function of these factors, in detail. The activity of these factors has been examined in various biological pathways. A list of proteins with activity in one or more pathways is provided in Table 1.

## 4. TGFβ Pathway Inhibitors

**Figure 2:**
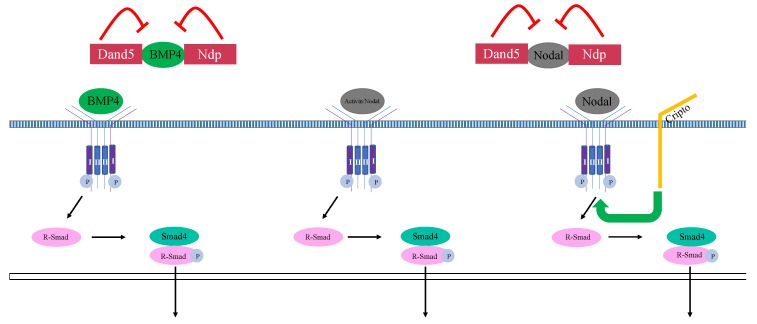
Extracellular regulation of mesendodermal gene expression. In this and subsequent figures, red boxes denote mesendoderm inhibitors. R-Smads refer to Smads1/5/8 or Smads2/3 for the BMP and Activin/Nodal pathways, respectively. TGFβ ligands (BMP4 and Activin/Nodal) bind the TGFβ receptor complex and activate signal transduction of the TGFβ signaling pathway. Dand5 (Coco) and Ndp (Norrin) physically associate with BMP and Activin/Nodal and inhibit signal transduction.

Several of the proteins identified as necessary for repression of mesoderm and/or endoderm in the ectoderm are inhibitors of the Activin/Nodal signaling pathway. One such inhibitor of mesendoderm expression in the ectoderm, Dand5 (Coco), belongs to the Cerberus/DAN/Gremlin superfamily. Members of this superfamily were originally identified as antagonists of BMPs [56,57,58]. The BMP signaling pathway, like the Nodal/Activin pathway, is a branch of the TGFβ signaling network; during development, BMP signaling specifies dorsal/ventral axis formation [59,60,61,62]. It was subsequently determined that some members of the Cerberus/DAN/Gremlin superfamily function as secreted inhibitors of both BMP and Activin/Nodal ligands, many of which are present in the endoderm and mesoderm [56,57,58]. In the ectoderm, Dand5, functions as a TGFβ ligand antagonist and, supplied maternally, is one of the earliest expressed antagonists of TGFβ signaling (Figure 2) [39]. In animal cap explants, Dand5 physically associates with both BMP4 and Xnr1, ligands of the BMP and Activin/Nodal pathways, respectively [39,63,64]. A population of pluripotent cells can be isolated from the animal pole of blastula stage *Xenopus laevis* embryos; this explant, called an “animal cap,” can be induced to differentiate into various tissue types [65]. Dand5 inhibits the mesoderm-inducing abilities of *Xnr1* misexpression as well as the ventralizing effects of *BMP4* misexpression [39]. The knockdown of *dand5* in animal caps leads to increased levels of phosphorylated Smad2 and increased expression of mesodermal markers [40]. Loss of Dand5 in whole embryos leads to increased endodermal marker expression at the expense of mesoderm; the knockdown of *dand5* in the presumptive mesoderm causes a reduction of the dorsal marker *chordin* [40]. Loss of Dand5 in whole embryos also leads to loss of Hoxb9, a spinal cord marker, suggesting a ventralizing effect [40]. Furthermore, these experiments demonstrate that Dand5 inhibits both the BMP and Activin/Nodal branches of the TGFβ signaling pathway and is necessary for repression of inappropriate mesendodermal gene expression in the ectoderm during gastrulation [39,40]. 

At early cleavage stages, another maternal TGFβ ligand inhibitor, Ndp (Norrin), is expressed in the prospective ectoderm [41]. Ndp is a secreted protein characterized by a cysteine-knot motif and was initially shown to function as a ligand in the Wnt signaling pathway [66,67]. Misexpression of *ndp* in animal caps represses Activin-mediated mesendoderm induction, and promotes neural fate, suggesting a repression of BMP signaling [41]. Co-immunoprecipitation experiments show that Ndp physically associates with the BMP pathway ligand, BMP4, and, in cell culture experiments, the Activin/Nodal pathway ligand, Xnr1 [41]. These studies suggest that Ndp inhibits TGFβ signaling via association with TGFβ ligands (Figure 2).

**Figure 3:**
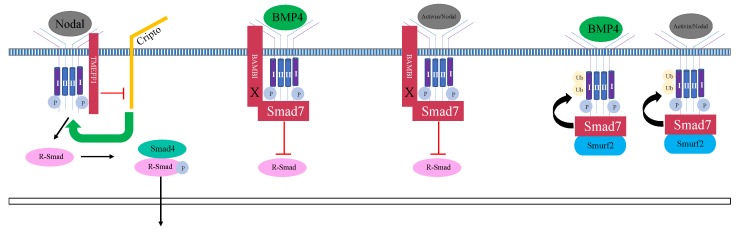
Transmembrane and cytosolic inhibition of mesendodermal gene expression. TMEFF1, a transmembrane protein, prevents the association between Cripto, a Nodal-pathway specific coreceptor, and the type I receptor. Smad7 inhibits TGFβ signaling by forming a complex with Smurf2, which subsequently induces the degradation of the type I and type II TGFβ receptors. BAMBI, another transmembrane protein, associates with Smad7 and the type I receptor and inhibits association between the type I receptor and R-Smads. The “X” indicates the lack of a serine/threonine intracellular kinase domain.

Another repressor of aberrant gene expression in the ectoderm is Tomoregulin-1 (TMEFF1), a transmembrane protein [42,68]. Misexpression of TMEFF1 suppresses Nodal- and Vg1- induced mesendoderm expression; TMEFF1 does not inhibit Activin-induced mesendoderm [42]. The association of Nodal with the type II and type I TGFβ receptors and subsequent phosphorylation of Smad2 depends on a Cripto family co-receptor [69]. While important for signal transduction of the TGFβ pathway upon Nodal ligand binding, Cripto has not been implicated in Activin signaling [69]. Cripto physically associates with the TGFβ type I receptor and promotes the association of Nodal and the type I receptor. TMEFF1 inhibits Nodal signaling by physically associating with the type I co-receptor and preventing the type I co-receptor from forming a complex with Cripto, an association necessary for signal transduction (Figure 3) [68].

**Figure 4:**
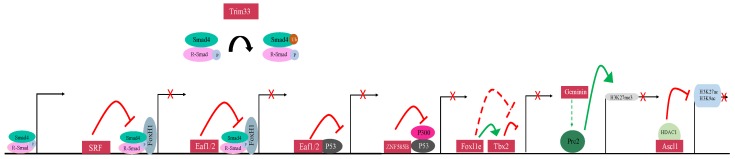
Nuclear regulation of mesendodermal gene silencing. Trim33 (Ectodermin) functions in the nucleus and, via ubiquitination, promotes the degradation of Smad4. At the transcriptional level, SRF prevents the association between FoxH1 and the Smad2-Smad4 complex, repressing Smad2 target genes. Eaf1/2 are repressors that inhibit Activin-mediated mesoderm induction via P53-dependent and P53-independent mechanisms. Eaf1/2 physically associates with P53, Smad2, Smad3, and FoxH1. ZNF585B (XFDL156) reduces the amount of P53 bound to P53 target sites and represses P53-induced mesodermal gene expression. FoxI1e is an activator that likely indirectly inhibits mesendodermal gene expression in the ectoderm. Tbx2, a T-box transcription factor, also represses mesendodermal gene expression. Geminin is a chromatin modifier that represses gene expression by recruiting the PRC2 complex. The PRC2 complex then trimethylates H3K27 to silence gene expression. Ascl1, another chromatin modifier, recruits HDAC1 to deacetylate H3K27 and H3K9, a mechanism that silences gene expression.

Another identified mechanism of mesoderm repression is via Smad4 inhibition. Smad4, an intracellular mediator of TGFβ signaling, forms a heteromeric complex with Smad1/5/8 or Smad2/3 in the BMP and Activin/Nodal pathways, respectively. In the ectoderm, Trim33 (Ectodermin), a RING-type ubiquitin ligase, physically associates with Smad4 (Figure 4) [43]. This association promotes the degradation of Smad4 [43]. As a downstream mediator of both BMP and Activin/Nodal signaling, degradation of Smad4 inhibits signaling in both pathways [70]. Loss of Trim33 in the prospective marginal zone of *Xenopus laevis* embryos expands the expression of endodermal markers, and loss of Trim33 in animal cap explants induces expression of mesodermal markers [43]. Misexpression of Trim33 in the prospective mesoderm represses mesodermal and ventral markers and increases the expression of neural markers [43]. These data show that Trim33 is necessary for inhibition of mesodermal and endodermal gene expression in the ectoderm.

An additional potent inhibitor of TGFβ signaling is Smad7, an “anti-Smad” [44]. Misexpression experiments in *Xenopus laevis* demonstrate that Smad7 is sufficient to reduce mesodermal markers in both the mesoderm and in Activin-treated animal cap explants [45]. Smad7 also inhibits the expression of ventral markers [45]. This indicates that Smad7 inhibits the Activin/Nodal and BMP pathways during gastrulation [44,45]. The molecular mechanisms through which Smad7 inhibits TGFβ signaling have been demonstrated in cell culture experiments. Smad7 physically associates with Smurf2 via a PPXY sequence in its linker region. Smad7 regulates the localization of Smurf2; the Smurf2-Smad7 complex translocates from the nucleus to the cytosol where it associates with a heteromeric type I and II receptor complex (Figure 3) [71]. Once associated with the TGFβ heteromeric complex, Smurf2 induces degradation of the TGFβ receptors via both proteasomal and lysosomal pathways [71]. These data from cell culture experiments provide insight into how Smad7 may function to inhibit mesodermal and endodermal gene expression during gastrulation. Smad6, another anti-Smad, blocks BMP signaling in *Xenopus laevis* [72,73,74]. Studies in *Xenopus laevis* have shown that *smad6* overexpression partially inhibits Activin-mediated mesoderm induction; however, the extent of this repression is not well studied [74].

BMP and Activin membrane-bound inhibitor (BAMBI) is a transmembrane protein with an extracellular domain that is similar to the BMP type I receptor but lacks an intracellular serine/threonine kinase domain [46]. Misexpression of BAMBI in *Xenopus laevis* animal caps is sufficient to repress the effects of both BMP4 misexpression and Activin treatment [46]. BAMBI inhibits TGFβ signaling by associating with the type I and type II receptor complex (Figure 3) [46]. BAMBI decreases the phosphorylation level of the type I receptor, and the dimerization of the type I receptor, a necessary component of the TGFβ signaling pathway [75]. Cell culture experiments show that BAMBI promotes the formation of a ternary complex composed of Smad7, BAMBI, and the BMP type I receptor (Figure 3) [47]. In this case, BAMBI does not induce Smurf-mediated receptor degradation [47]. Instead, the Smad7-BAMBI-type I receptor complex inhibits the physical association between the type I receptor and R-Smads [47].

Regulation of TGFβ signaling also occurs downstream of the Smad2-Smad4 complex. The serum response factor (SRF) belongs to the MADS-box family of transcription factors [76]. MADS-box transcription factors contain a conserved 56 amino acid MADS-box which confers DNA-binding activity [76]. During gastrulation, SRF transcripts are present predominantly in the ectoderm of *Xenopus laevis* embryos, with low levels detected in the marginal zone and no expression of SRF detected in the endoderm [48]. Loss-of-function studies demonstrate that SRF is necessary for the repression of mesodermal markers in the ectoderm. Consistently, expression of a dominant-negative form of SRF in animal cap explants of *Xenopus laevis* embryos leads to ectopic expression of mesoderm [48]. The knockdown of SRF in whole embryos leads to an expansion of mesodermal markers towards the ectoderm [48]. An investigation into the mechanism of action of SRF revealed that SRF represses mesoderm in the developing embryo by inhibiting the association between Smad2 and Fast1 [49]. Fast-1 (FoxH1) is a winged-helix transcription factor that physically associates with Smad2 and functions as a co-activator to turn on target genes [10,49]. Through this mechanism, SRF functions in the ectoderm to limit mesendoderm expression (Figure 4) [48]. 

Initially identified as tumor suppressors, Eaf1/2 have also been shown to inhibit phosphorylated Smad2 activity during germ layer specification [50,77]. Eaf1/2 are ELL-associated factors that were initially shown to function as antagonists of Wnt/β-catenin signaling during development [78]. During development, Wnt/β-catenin plays an important role in establishing the dorsoventral and anteroposterior axes of the embryo [79]. Consistent with this, misexpression experiments in zebrafish demonstrate that Eaf1/2 increase dorsal markers during gastrulation [78]. Eaf1/2 function as transcriptional repressors to inhibit Wnt/β-catenin signaling [78]. Eaf1/2 were later studied for their role in germ layer specification during gastrulation. Misexpression of Eaf1/2 reduce the expression of mesodermal and endodermal markers in zebrafish embryos. Loss of function experiments show that the ectodermal marker foxi1 decreases in *eaf1/2* morphants [50]. Studies in cell culture show that Eaf1/2 co-localize and physically associate with Smad2. Chromatin immunoprecipitation experiments reveal that Eaf1/2 occupy promoters of TGFβ targets, suggesting that Eaf1/2 function as transcriptional repressors, as they function in the Wnt/β-catenin pathway, to repress the mesoderm-inducing activities of the Activin/Nodal branch of the TGFβ signaling pathway, and specifically the function of Smad2 (Figure 4) [50]. 

## 5. P53 Inhibitors in Mesoderm Repression

P53 has been shown to induce mesoderm when overexpressed in animal cap explants of *Xenopus laevis* embryos; phosphorylated P53 physically associates with phosphorylated Smad2 to induce mesodermal marker expression [24,26]. Knockdown of P53 in e*af1/2* morphant zebrafish embryos reduces levels of mesodermal marker expression, suggesting that Eaf1/2 may partially suppress mesodermal markers through a P53-dependent mechanism [50]. However, Eaf1/2 are still able to repress TGFβ targets whose induction is independent of P53 function: Eaf1/2 suppress P53-independent targets of TGFβ signaling in P53 mutant embryos [50]. Studies also show that Eaf1/2 physically associate with P53 and repress P53 response elements and P53-required TGFβ Luciferase reporters (Figure 4) [50]. These data suggest that Eaf1/2 function at the transcriptional level to repress P53-mediated mesoderm induction [50].

Another inhibitor of P53-induced mesoderm expression is ZNF585B (XFDL156). ZNF585B is a zinc finger nuclear factor present in *Xenopus laevis* embryos in the ectoderm during early gastrulation [51]. Misexpression experiments in animal cap explants reveal that ZNF585B specifically represses mesodermal, and not endodermal, marker expression, suggesting that ZNF585B does not function as a TFG pathway signaling inhibitor [51]. This led to the discovery that ZNF585B functions as a P53 inhibitor; it physically associates with P53 and decreases the level of P53 binding to target promoter sites (Figure 4) [51]. The C-terminus of P53 is referred to as the regulatory domain (RD); removal of this domain increases the mesoderm-inducing ability of P53 [26,27]. The deletion of the RD reduced the ability of ZNF585B to effectively inhibit mesoderm induction via P53, indicating that the P53 RD is necessary for ZNF585B suppressor activity [51].

## 6. Additional Transcriptional Regulators of Inappropriate Germ Layer Expression

The transcription factor, FoxI1e (Xema) is required for the suppression of mesendodermal expression in the ectoderm during gastrulation (Figure 4). The Fox transcription factors are characterized by a conserved winged-helix DNA-binding domain, and many are present during early embryogenesis [80]. Misexpression of FoxI1e represses both Activin and FGF-mediated mesoderm induction, suggesting that FoxI1e may not act directly, or at least not only, as a TGFβ pathway inhibitor [52]. FoxI1e functions as a transcriptional activator during early development [52]. Expression of a chimeric protein consisting of the FoxI1e coding region fused to an Engrailed repressor domain induces mesodermal marker expression in animal cap explants, suggesting that repression of FoxI1e transcriptional targets is sufficient to induce mesoderm; knockdown of foxi1e similarly results in ectopic mesodermal marker expression [52]. Misexpression of FoxI1e in the endoderm induces the expression of both epidermal and neural ectodermal markers [81]. FoxI1e also plays a role in the spatial regulation of ectodermal cells: loss of FoxI1e causes ectodermal cells to lose adhesive properties and relocate to other germ layers [81].

As a transcriptional activator, it is likely that FoxI1e suppresses mesoderm indirectly, possibly through activation of a transcriptional repressor responsible for suppressing mesodermal and endodermal gene expression in the ectoderm. *tbx2*, a gene encoding a T-box family transcription factor, has been identified as a target of FoxI1e (Figure 4) [53]. The structure of T-box proteins is conserved across five subfamilies [82]. All T-box proteins contain a highly conserved region of 180–200 amino acids, called the T-box, which confers DNA-binding specificity [83]. T-box proteins can function as activators or repressors of transcription [84,85]. Like *foxi1e*, *tbx2* is expressed at high levels in the animal pole during gastrulation [53]. Also, like FoxI1e, Tbx2 represses Activin and FGF-mediated mesoderm induction [53]. However, unlike FoxI1e, Tbx2 functions as a transcriptional repressor [53]. Misexpression experiments show that Tbx2 also represses ventral fate in animal cap explants and induces expression of the “default” dorsal fate—neural tissue [53]. Ectopic expression of Tbx2 in the marginal zone represses mesodermal and ventral markers [86]. To further demonstrate the repressive effect of Tbx2, the promoter region of *bix4*, a target of the T-box transcription factors Brachyury and VegT, was fused to a Luciferase reporter gene. Bix4-promoter/Luciferase experiments suggest that Tbx2 requires the T-box sites on the Bix4 promoter for repression [53]. The ability of *tbx2* to repress both BMP and Activin/Nodal pathways suggests that Tbx2 may function through the TGFβ pathway and/or TGFβ target gene inhibition; however, the ability of Tbx2 to repress FGF-mediated mesoderm induction suggests that Tbx2 may additionally repress transcription through TGFβ-independent mechanisms.

## 7. Epigenetic Suppressors of Mesendoderm

In addition to the TGFβ pathway inhibitors and transcription factors described above, epigenetic modifiers have been implicated in mesoderm and endoderm suppression. Geminin, a nuclear protein, was initially identified as a regulator of DNA replication [87]. During gastrulation, Geminin promotes the expression of neural markers at the expense of epidermal markers [88]. Misexpression of *geminin* suppressed both mesodermal and endodermal markers in Activin- and FGF- treated animal cap explants. [54]. The knockdown of Geminin expands the expression patterns of mesodermal and endodermal genes; however, the knockdown of Geminin in the animal pole of whole embryos is insufficient to induce expression of mesodermal and endodermal markers [54]. Geminin has been shown to repress transcription through the Polycomb Repressive Complex (PRC2); PRC2 is a cluster of proteins that act as an epigenetic modulator to suppress transcription and has been implicated in many biological processes including development (Figure 4) [89,90]. Specifically, PRC2 functions as a methyltransferase to trimethylate H3K27 and repress transcription [91]. PRC2 is comprised of the proteins Ezh2, Suz12, and Eed [92]. Knockdown of either Suz12 or Ezh2 inhibits the repressive effects of Geminin misexpression, suggesting that an intact PRC2 is necessary for Geminin function [54]. 

Another chromatin modifier implicated in repression of inappropriate gene expression in the ectoderm is Ascl1. Ascl1 has been shown to neuralize mouse embryonic fibroblasts [93]. In *Xenopus laevis*, Ascl1 inhibits VegT-mediated mesendoderm induction, but not Activin/Nodal-mediated mesoderm induction. During gastrulation, VegT directly activates various Nodal-related mesendoderm inducers [25,94]. Ascl1 is expressed maternally, and during gastrulation is detected at high levels in the ectoderm and at lower levels in the marginal zone [55]. Experiments in both mammalian cell culture and *Xenopus laevis* embryos reveal that Ascl1 functions to recruit HDAC1 to reduce H3K27 acetylation, a marker of actively transcribed promoters (Figure 4) [55,95]. Microinjection of VegT increases levels of H3K27ac and H3K9ac (also a hallmark of active promoters) on mesodermal and endodermal genes such as Nodal, and these levels are reduced by misexpression of Ascl1 [55,95,96].

## 8. Conclusions

Vertebrate germ layer formation and patterning is a complex process that involves the suppression of multiple signaling pathways. Highlighted in this review are several inhibitors of TGFβ signaling, necessary for repression of mesendodermal gene expression in the presumptive ectoderm. These antagonists function at many steps in the pathway, from ligand-receptor complex formation to TGFβ-mediated regulation of transcription. The requirement for additional transcriptional repressors and chromatin modifiers demonstrates that inhibition at multiple network nodes is necessary to restrict mesendoderm during gastrulation. 

The restriction of mesendodermal gene expression in the animal pole and inhibition of BMP signaling in the dorsal ectoderm during early development are sufficient to give rise to neural tissue. These instances of gene repression are examples of a common theme found throughout developmental biology, whereby localized repression of gene expression within the developing embryo gives rise to “zones of plasticity” allowing distinct cell fates to arise. A somewhat analogous process occurs during early post-implantation stages of mouse development. Initially *nodal* is expressed throughout the epiblast and is necessary for proximal-distal patterning [97]. Subsequently, during gastrulation, the anterior epiblast, fated to become ectoderm, exhibits little Nodal signaling due to localized repression by multiple extracellular Nodal antagonists [98]. Studies in cell culture show that inhibition of Nodal signaling specifies a transient ectodermal progenitor population that can give rise to either neural or epidermal ectodermal fates [99]. This review highlights the process by which multiple factors, via inhibition at multiple signaling nodes, specify a region devoid of mesendoderm-inducing and ventralizing signals during gastrulation. 

## Figures and Tables

**Table 1:** Inhibitors of mesoderm in *Xenopus laevis* ectoderm.

Gene Name	Blocks Mesoderm via Inhibition of TGFβ Signal Transduction	Blocks Mesoderm via Alternative Pathway	Additional Comments	References
Dand5 (Coco)	+		Blocks via ligand inhibition	[39,40]
Ndp (Norrin)	+		Blocks via ligand inhibition	[41]
Tomoregulin-1 (TMEFF1)	+		Inhibits Cripto/receptor complex	[42]
Trim33 (Ectodermin)	+		Promotes degradation of Smad4	[43]
Smad7	+		Inhibitory Smad	[44,45]
BAMBI	+		Inhibits receptor/Smad association	[46,47]
Serum Response Factor (SRF)	+		Inhibits FoxH1/Smad2 association	[48,49]
Eaf1/2	+	+	Associates with Smad2 and P53	[50]
ZNF585B (XFDL156)		+	P53 inhibitor	[51]
FoxI1e		+	Transcriptional activator	[52]
Tbx2		+	Transcriptional repressor	[53]
Geminin		+	PRC2 dependent	[54]
Ascl1		+	Recruits HDAC1	[55]
